# Synthesis, Characterization and Application of Biobased Unsaturated Polyester Resin Reinforced with Unmodified/Modified Biosilica Nanoparticles

**DOI:** 10.3390/polym15183756

**Published:** 2023-09-14

**Authors:** Hifa Salah Adeen Embirsh, Ivana Stajčić, Jelena Gržetić, Ivana O. Mladenović, Boban Anđelković, Aleksandar Marinković, Marija M. Vuksanović

**Affiliations:** 1 Faculty of Technology and Metallurgy, University of Belgrade, 11120 Belgrade, Serbia; yhaifa23@yahoo.com (H.S.A.E.); marinko@tmf.bg.ac.rs (A.M.); 2 Department of Physical Chemistry, “VINČA” Institute of Nuclear Sciences—National Institute of the Republic of Serbia, University of Belgrade, 11351 Belgrade, Serbia; ivana_r@vin.bg.ac.rs; 3 Military Technical Institute, 11030 Belgrade, Serbia; jrusmirovic@tmf.bg.ac.rs; 4 Institute of Chemistry, Technology and Metallurgy, National Institute of the Republic of Serbia, University of Belgrade, 11000 Belgrade, Serbia; ivana.mladenovic@ihtm.bg.ac.rs; 5 Faculty of Chemistry, University of Belgrade, Studentski Trg, 12-16, 11158 Belgrade, Serbia; aboban@chem.bg.ac.rs; 6 Department of Chemical Dynamics and Permanent Education, “VINČA” Institute of Nuclear Sciences—National Institute of the Republic of Serbia, University of Belgrade, 11351 Belgrade, Serbia

**Keywords:** polymer–matrix composites (PMCs), recycling, biosilica, mechanical properties

## Abstract

This paper presents sustainable technology for environmentally friendly composite production. Biobased unsaturated polyester resin (b-UPR), synthesized from waste polyethylene terephthalate (PET) glycosylate and renewable origin maleic anhydride (MAnh) and propylene glycol (PG), was reinforced with unmodified and vinyl-modified biosilica nanoparticles obtained from rice husk. The structural and morphological properties of the obtained particles, b-UPR, as well as composites, were characterized by Fourier-transform infrared spectroscopy (FTIR), nuclear magnetic resonance spectroscopy (NMR), scanning electron microscopy (SEM), and transmission electron microscopy (TEM) techniques. The study of the influence of biosilica modification on the mechanical properties of composites was supported by hardness modeling. Improvement of the tensile strength of the b-UPR-based composite at 2.5 wt.% addition of biosilica modified with vinyl silane, named “b-UPR/SiO_2_-V” composite, has been achieved with 88% increase. The thermal aging process applied to the b-UPR/SiO_2_-V composite, which simulates use over the product’s lifetime, leads to the deterioration of composites that were used as fillers in commercial unsaturated polyester resin (c-UPR). The grinded artificially aged b-UPR composites were used as filler in c-UPR for the production of a table top layer with outstanding mechanical properties, i.e., impact resistance and microhardness, as well as fire resistance rated in the V-0 category according to the UL-94 test. Developing sustainable composites that are chemically synthesized from renewable sources is important from the aspect of preserving the environment and existing resources as well as the extending their life cycle.

## 1. Introduction

Polymers synthesized from natural resources have attracted attention from both the scientific community and industry due to environmental concerns regarding the exploitation of oil and the accumulation of non-degradable polymeric waste. In order to address these issues, researchers have been focusing on the production of biobased materials as well as finding the most efficient recycling route for various commercial polymers [1,2]. Poly(ethylene terephthalate) (PET) is among the most commonly and widely used non-degradable but recyclable polymers, with applications expanding from products for everyday consumption, such as plastic bottles, to fine electronic sensors [3,4,5,6]. Chemical recycling includes reactants or solvents to induce depolymerization process through the breaking of chemical bonds [7,8,9]. Chemical recycling offers the possibility of depolymerization and modification with the outcome of monomers and novel materials [10,11,12]. Depolymerized PET yields various products (oligoesters) that can be used as precursors to create new value-added polymers such as saturated and unsaturated polyesters, polyisocyanurate, and polyurethanes [13,14]. Polyurethanes (from recycled PET) have also been reported in the literature as new binders in environmentally friendly rocket fuels. Because they have comparable performance characteristics such as thermal stability and mechanical properties, these new binders can successfully replace traditional binders in existing rocket fuels [15,16,17]. Unsaturated polyester resins obtained by PET glycolysis using propylene glycol and maleic anhydride showed potential for reuse as versatile reactants in the syntheses of novel polymers, reducing the consumption of petroleum-based monomers [18,19]. Furthermore, propylene glycol and maleic anhydride have been successfully synthesized from renewable sources, making the entire process of PET depolymerization biobased [20,21].

The development of nanotechnology increases the production of nanocomposites with controllable functional properties [22,23]. Silica nanoparticles are commonly used as reinforcement in polymer nanocomposites due to their increased impact and tensile strength, as well as their thermal stability [24,25]. However, due to their inorganic/organic surface incompatibility, silica particles require chemical surface functionalization with organic modifiers, in order to improve particle dispersion in a polymer matrix [26]. Various types of silanes are proven to be very efficient for reducing the surface hydroxyl group content in silica particles [27]. Siot et al. investigated the influence of unmodified and poly(methyl methacrylate) (PMMA)-modified silica on mechanical properties [28]. They showed that particle dispersion processes and modifications with PMMA have a great influence on yield stress and Young’s modulus. Venkatesan et al. determined that tensile strength increased with nanosilica loading in poly(butylene adipate-co-terephthalate)-based nanocomposite [29]. By varying amounts, they have established that an increase in tensile strength of nearly 100% could be achieved with 10 wt.% of nanosilica. Picu et al. [30] functionalized silica nanoparticles with phenylazide, enabling the control of mechanical properties by applying ultraviolet (UV) radiation or heat on epoxy nanocomposite. Recently, a review presented different types of nanosilica synthesis and its advantageous influence on the mechanical performance of polyester resins [31]. Surface modification of silica particles was often applied to improve interfacial compatibility and dispersed in the matrix, which is an important factor in strengthening and toughening the matrix material [31].

However, in most of the studies, silica synthesized using tetraethyl orthosilicate was used, a compound that originates from oil, whereas silica can be obtained from renewable sources, such as rice and corn husk [32,33,34]. Waste rice husk (RH) represents an environmental problem in undeveloped countries due to open-air incineration, which causes high pollution [35]. Using a low-cost procedure of chemical treatment and calcination of rice husk, silica-rich powder is produced [36,37]. The latest studies show the influence of biosilica on the mechanical performance of epoxy-based nanocomposites. Neopolean et al. showed that the addition of bagasse biosilica and Opuntia natural fiber increased epoxy tensile properties by 100% [38]. Balaji et al. studied the influence of rice husk silica and pear fibers on dynamic mechanical properties. They revealed that the addition of RH biosilica leads to an increase in storage modulus and a reduction in loss factor [39]. Alshahrani et al. showed that the addition of RH biosilica increases the tensile and flexural properties of epoxy resin [40]. In our previous study, commercial nanosilica was used as reinforcement for unsaturated waste PET-based resin, resulting in a mechanical performance increase [13]. However, according to the authors’ knowledge, there are no studies regarding the RH biosilica influence on the mechanical properties of unsaturated polyester resins, especially waste PET-based resins. By incorporating RH biosilica, an entire nanocomposite can be processed out of biobased starting constituents.

The goal of this work was to develop nanocomposites, which include the principles of green chemistry achieved through three steps: (1) synthesis of b-UPR from biobased reactants, i.e., PG and MAnh, and raw material based on recycled PET (depolymerization was carried out using biobased PG, yielding a hydroxy-terminated glycolyzate); (2) production of a nanocomposite based on b-UPR reinforced with unmodified and modified biosilica (methacryl, vinyl, and two-stage amino silane and biodiesel obtained from soybean oil). The structural, morphological, and mechanical properties of the b-UPR matrix and the produced composites were investigated. Using the standard Vickers hardness method and Crawford’s model [41] helped in predicting the response of the hardness value of the hybrid composite, i.e., b-UPR/biosilica, with load and dwell time variations. (3) The third step concerned the extension of the composite’s life cycle. To obtain table top layers, artificially aged b-UPR composites were used as ground fillers in new composites made from commercial c-UPR. The resulting materials exhibited exceptional mechanical properties as well as excellent fire resistance (non-combustible material, category V-0).

## 2. Materials and Methods

### 2.1. Basic Materials and Syntheses of Composites

#### 2.1.1. Preparation and Modification of the Silica Particles

Rice husk (from Levidiagro in Kočani, North Macedonia), which was used to make biosilica particles, was washed, dried, and powdered before being treated with 10% sulfuric acid (Zorka Šabac, Serbia) at 80 °C for 3 h. The shell was washed up to pH~7 with distilled water, dried at 105 °C, and then torched. After heating the black material, white biosilica particles were formed. A detailed description of biosilica preparation is given in the literature [42].

Surface modification of the obtained silica particles was performed using three types of silanes: 3-trimethoxysilylpropyl methacrylate (MEMO), trimethoxyvinylsilane (TMEVS), and 3-aminopropyltrimethoxysilane (APTES) were purchased from Sigma-Aldrich, Darmstadt, Germany. Additional chemicals (p.a. quality) purchased from Sigma Aldrich, Darmstadt, Germany: ethanol, methyl methacrylate, 2-butanone peroxide (methyl ethyl ketone peroxide; MEKP), copper(II) nitrate trihydrate (Cu(NO_3_)_2_·3H_2_O), glycerol, conc. HCl (36%), conc. Sulfuric acid (98%), γ-Al_2_O_3_, styrene, tetrabutyl titanate (TBT), xylene (mixture of 1,2-, 1,3- and 1,4-dimethylbenzene), cobalt octoate (Co-oct), hydroquinone (HQ), toluene, p-toluene sulfonic acid, pyridine, tetrahydrofuran (THF), acetonitrile (MeCN), fructose, hydrogen peroxide 30%, sodium bisulfite (NaHSO_3_), acetic anhydride, potassium hydroxide, iodine monochloride, acetic acid, starch water soluble, carbon tetrachloride, sodium thiosulfate, potassium iodide, NaHCO_3_, CaCl_2_, and MgSO_4_. Wheat straw was provided by local farmer near to Belgrade (village Bečmen, Surčin, Serbia). TiO_2_ P-25 (Degussa) with 70% anatase and 30% rutile, a surface area of 50 m^2^/g, and a particle size of 25 nm were used in photocatalytic experiments.

Modification with MEMO and TMEVS—In a three-necked dry glass flask (100 mL), equipped with a magnetic stirrer, a reflux condenser, a thermometer, and a nitrogen inlet tube, was added 20 mL of toluene and 6 g of SiO_2_ with high-speed stirring (500 rpm) for 15 min. For achievement of good biosilica dispersion in toluene, ultrasound was applied for 15 min. The addition of 2.5 mL of MEMO silane and modification was performed at 70 °C for 24 h providing gentle mixing (150 rpm). For achievement of good biosilica dispersion in toluene, ultrasound was applied for 5 min prior to heating. After the reaction was completed, the mixture was filtered using vacuum filtration and washed with toluene and ethanol to remove the residual unreacted components and left to dry at 60 °C for 24 h. Modification of the particles with TMEVS was performed in the same way as with MEMO.

Modification with APTES and BD—First step: Biosilica was modified with APTES according to the procedure given for modification with MEMO and TMEVS. Amino number of 280 μmol g^−1^ reflects appropriate amino reactivity able to create an amide bond with a biodiesel of soybean oil. Second step: amino-terminated biosilica particles’ modification with BD. The synthesis of biodiesel from soybean oil was performed according to Rusmirović et al. [43]. The obtained amino-modified biosilica was placed in a three-necked dry glass reactor, and 50 mL of tetrahydrofuran (THF) and 3 g of soybean fatty acid methyl ester were added afterwards. The reaction was carried out for 12 h at 25 °C, after which the mixture was heated to 60 °C and kept for another 2 h at the set temperature. The product was isolated by vacuum filtration, washed twice with toluene and absolute ethanol, and dried for 12 h at 40 °C.

#### 2.1.2. The Synthesis of b-UPR from Biobased Reactants

For the production of unsaturated polyester resin, waste PET collected from soft drink bottles was used. The PET waste was cut into small pieces (approximately 0.5 × 0.5 cm) and washed with detergent and ethanol to remove all traces of impurities and residual adhesives. Fascat 4100 (butylstannoic acid) was produced by PMC Organometallix, The Netherlands, NZ Hoofddor, where PET:PG = 2:1 wt. ratio (130 g, 0.67 mol, waste PET:65 g PG, 0.86 mol). The synthesis of b-UPR was performed according to the procedure described in our previous paper [44]. Synthesis of MAnh and PG from biobased resources: γ-Al_2_O_3_ was impregnated with Cu(NO_3_)_2_·3H_2_O water solution to obtain catalysts with ~20 wt.% copper loading. The solvent was removed by air bubbling; the samples were then heated at 110 °C for 12 h and calcined at 700 °C for 5 h [21]. The hydrogenolysis reaction of glycerol to give 1,2-propanediol (1,2-PG) was carried out in a stainless-steel autoclave (500 mL, Parr Company, Hillsboro, OR, USA) equipped with a stirrer, thermocouple, pressure gauge, and sampling tube. The detailed procedure is given in the literature [45]. The obtained PG was purified by vacuum distillation, and purity was confirmed by the results from FTIR, and elemental analysis, and refractive index determination, according to ASTM D 1218-02.

The detailed description of maleic anhydride (MAnh) synthesis was performed using air as an oxidant with the help of MnO_2_/Cu(NO_3_)_2_ as a catalyst following the procedure given by Wenlong et al. [20].

After glycolysis (synthesis of PET glycolizate by the classical method) [43], the reaction mixture was cooled to 90 °C, the Dean–Stark water separator was assembled, and MAnh (59.0 g, 0.60 mol) and HQ (0.02 g) were added to the flask. The mixture was heated at 115 °C for 1 h. Afterward, a continuous temperature increase was achieved at a heating rate of 15 °C/h. When the reaction mixture temperature reached 150 °C, xylene (5.5 g, 3 wt.% with respect to the reactant weight) was added to provide water removal by binary azeotrope, ensuring in this manner a driving force for reaching the end-point of reaction at 210 °C. After completion of the reaction (10.5 mL of water separated), the obtained resin was cooled to 120 °C, and 0.02 g HQ in 0.2 mL of absolute ethanol was added. Subsequently, vacuum distillation (water pump) was carefully applied for 1 h to remove low boiling compounds with a slow temperature decreasing to 90 °C. The hot resin was slowly poured out into contain with styrene (final dilution was set at 40 wt.% styrene addition) with constant slow mixing (50 rpm) to ensure homogeneity of the b-UPR. The color of the composite resin is faintly greenish.

#### 2.1.3. The Preparation of a Nanocomposite Based on b-UPR Reinforced with Unmodified and Modified Biosilica

b-UPR was used as a polymer matrix, and biosilica particles were used as composite reinforcement. Composites based on b-UPR and either unmodified or modified biosilica particles were subjected to a homogenization procedure to obtain uniform nano-filler dispersion. The contents of the reinforcement used in the preparation of composites are 1, 2.5, and 5 wt.%. biosilica particles were dispersed in b-UPR using a laboratory planetary mixer adapted to vacuum evacuation of gaseous bubbles from processing material (10 min). After that period, the initiator 1 wt.% MEKP and 0.5 wt.% cobalt-octoate were added, and homogenization (200 rpm) for 2 min under vacuum was performed to obtain a homogeneous pasty dispersion that was immediately poured into molds. The composites were first cured at room temperature for 24 h before being placed in an oven at 65 °C for 4 h and 80 °C for an additional 5 h.

#### 2.1.4. The Life Extension of the Composite

For preparation of table top composites, commercial UPR (c-UPR, i.e., DEPOL IN 200 M) was kindly provided by Axyntha Šabac, Serbia; antifoam agent BYK 054, BYK-Chemie, Altana, GA, USA; UV stabilizer Tinuvin 327, 2-(2′-Hydroxy-3′,5′-di-tert-butylphenyl)-5-chlorobenzotriazole, Rianlon, Duesseldorf, Germany; soybean oil and yellow pigment paste were donated by Interhem Company, Serbia; aluminum (III) hydroxide Alolt 2AF was purchased from MAL Hungarian Aluminum, Ajka, Hungary. Calcium hydroxide was provided by Centrohem, Belgrade, Serbia.

To achieve a consistent dispersion, composites manufactured from 33 wt.% commercial c-UPR, 7.9 wt.% ground aged composites based on b-UPR (with 5 wt.% SiO_2_-V), 0.1 wt.% BIK 054, 2 wt.% ethyl acrylate, 2 wt.% pigment paste, and 55 wt.% Al(OH)_3_ were homogenized. The second upper table product contains: 33 wt.% c-UPR; 19.9 wt.% ground aged composites based on b-UPR (with 5 wt.% SiO_2_-V); 0.1 wt.% BIK 054, 3 wt.% ethyl acrylate; 2 wt.% pigment paste, and 42 wt.% Al(OH)_3_. A laboratory planetary mixer fitted for the vacuum evacuation of gas bubbles from the material to be processed was used to homogenize the mixture. After mixing, the initiator (1 wt.% MEKP) and 0.5 wt.% cobalt-octoate are added, and the mixture is homogenized (50 rpm) for 2 min under vacuum to produce a homogenous, pasty dispersion that is immediately poured into molds.

### 2.2. Characterization Methods

Methods of characterization of biosilica nanoparticles before and after surface modification, b-UPR, obtained composites, and the table top are given in the next text.

^1^H and ^13^C NMR spectra were recorded in the CDCl_3_ on a Bruker Avance III 500/125 MHz, USA equipped with a broad-band direct probe at 500 MHz for ^1^H NMR and 125 MHz for ^13^C NMR.

The Wijs method was used to calculate the iodine value. Elemental analyses (C, H, and O) were performed by standard micro methods using an ELEMENTAR Vario EL III CHNS/O analyzer, GmbH. The gel time of the samples was determined according to ASTMD 2471-99.

To analyze the structural properties of the obtained glycolyzed product and synthesized plasticizers, silica particles, and composites, Fourier-transform infrared spectroscopy was used (ATR-FTIR Nicolet™ IS™ 10 Thermo Scientific, Waltham, MA, USA).

The hydroxyl number (HV) and acid number (AV) were determined using standard procedures (ISO 4326:1992) and (ASTM D3644) [43]. Determination of amino group: The amount of amino group was determined via “back” titration [46].

Characterization of the morphological properties of the obtained silica particles was carried out using a field emission scanning electron microscope (FE-SEM) Mira3 Tescan set to 20 kV, and a Philips CM12 transmission electron microscope (Philips/FEI, Eindhoven, The Netherlands) equipped with the digital camera SIS MegaView III (Olympus Soft Imaging Solutions, Münster, Germany). The silica particles were analyzed using energy-dispersive spectroscopy (EDS), Oxford Inca 3.2 peak, and a Jeol JSM 5800 (USA) scanning electron microscope running at 20 kV. A JEOL JSM6610LV SEM was used to provide microgrpahs of b-UPR composites.

Thermal analysis was performed using an SDT Q-600 simultaneous TG/DTG-DSC instrument (TA Instruments, New Castle, DE, USA). The samples were heated in a standard alumina sample pan from room temperature to 300 °C. Measurements were conducted at heating rates of 10 °C/min under nitrogen at a flow rate of 0.1 dm^3^/min.

To determine the microhardness of the composite materials, the Vickers method was used (model “Leitz Kleinert Prufer DURIMET I”, Oberkochen, Germany) with an applied load of 4.9 N and a holding (dwell) time of 25 s. Three punctures were made on each sample and the samples were recorded on an optical microscope (Carl Zeiss—Jena, NU2, Germany) [47]. For the Vickers hardness test, tree indentation load levels (25 p (0.2452 N), 50 p (0.4903 N), and 100 p (0.9806 N)) were selected with each tested with eight levels of holding time (0, 10, 20, 30, 40, 50, 100, and 300 s). The diagonal size was measured 100 s after the indent was made. The reduced time (delayed ratio) is calculated as the ratio of the delayed time of measurement to the dwell time [41,48]. In this case, reduced times were 0, 0.1, 0.2, 0.3, 0.4, 0.5, 1, and 3. For fitting hardness data, Crawford’s hardness model was used [41,48].

The Charpy method was used to test the toughness of composite in accordance with the standard EN ISO 179-1/1fU (EN ISO 179-1:2000) using the device Zwick & Co. KG. Germany [49]. Dynamic-mechanical analysis of cured composites was performed using the Modular Compact Rheometer MCR–302 (Anton Paar GmbH, Ashland, VA, USA) in the torsion deformation mode [50]. On a High-Speed Puncture Impact testing machine, the impact resistance of table top and floor material based on c-UPR composites was investigated (HIDROSHOT HITS-P10, Shimadzu, Kyoto, Japan) [51].

To accelerate aging, the composite samples were thermally aged in oven at different temperatures: 85 °C, 100 °C, 115 °C, 130 °C, and 175 °C. The total amount of time spent aging is 200 days. The samples were placed in the furnace once the temperature reached the required level and remained constant [52].

The UL-94 vertical test was carried out using tubes made of table top and floor material based on recycled b-UPR composites [53].

## 3. Results

### 3.1. Characterization of Raw Materials, Resins, and Composites

#### 3.1.1. Analysis of Raw Materials

**Figure 1:**
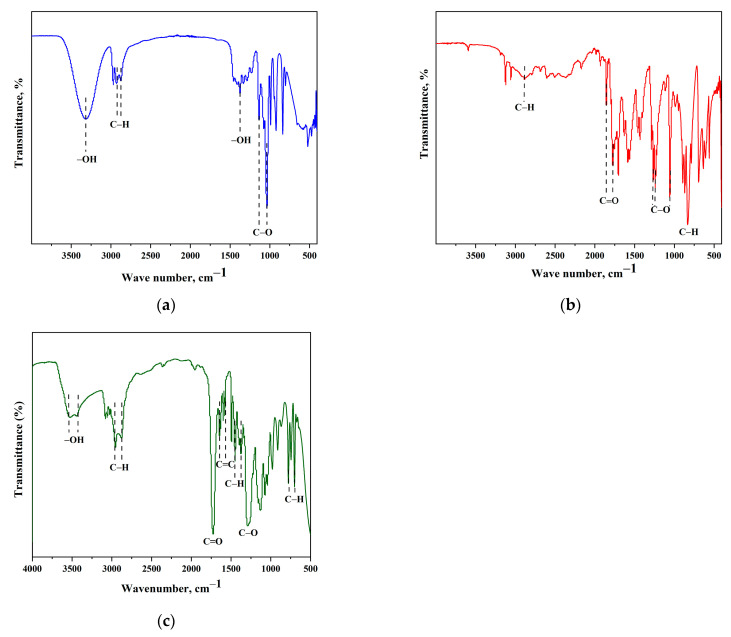
FTIR spectrum of synthesized: (**a**) PG, (**b**) MAnh and (**c**) b-UPR resin [58].

Purity determination was performed using GC analysis of PG (Gas chromatograph 7890A GC System, Agilent Technologies, Santa Clara, CA, USA): 98.6% elemental analysis (calculated, %): C, 47.35; H, 10.60; O, 42.05 elemental analysis (experimental, %): C, 47.58; H, 10.58; O, 41.99 Index of refraction (n_20_) = 1.4337 (lit. value 1.4329). The FTIR spectra of synthesized PG MAnh, and b-UPR resin are given in Figure 1, respectively. The stretching vibration of the O–H group is responsible for the absorption peak at 3316 cm^−1^ in the PG spectrum, while the sharp peaks at 2922 cm^−1^ and 2881 cm^−1^ belong to the C–H group stretching vibration [54]. Additionally, the O–H bending vibration was observed at 1374 cm^−1^. Secondary and primary C–O stretching appeared at 1129 cm^−1^ and 1030 cm^−1^, respectively. In the FTIR spectrum (Figure 1b), aliphatic –CH stretch appeared at 2880 cm^−1^. Asymmetric and symmetric C=O stretching was observed at 1860 cm^−1^ and 1776 cm^−1^, respectively [55]. Peaks in the region 1269–1060 cm^−1^ relate to C–O from cyclic anhydride [56]. The C–H deformation vibration of vinyl group is observed at 820 cm^−1^ [57].

The FTIR spectrum of synthesized b-UPR resin is given in Figure 1c, while the physicochemical characteristics of b-UPR are given in Table 1, respectively. The viscosity of the b-UPR, 60 wt.% styrene solutions, was measured at 25 °C using a Ford viscosity cup 4 (ASTM D1200). The calculated number average molar mass (Mn) was 3032 g/mol.

The gel time of b-UPR, as determined by the cure exotherm, was ~15 min, while that of c-UPR composites used for the table top was ~20 min. The viscosity of a 60 wt.% solution of b-UPR in styrene measured with the Ford viscosity cup ranged from 85 to 93 s. The short gel time and low viscosity of the synthesized UPR resins allow them to be used in practical, i.e., commercial, applications. The FTIR spectrum of b-UPR is given in Figure 1c.

The wide band at 3432–3528 cm^−1^ corresponds to the stretching vibrations of the OH groups from the residual reactants. The band at approximately 2981 cm^−1^ originates from the vibration of stretching aliphatic C–H bonds from the methyl and methylene groups. The peak corresponding to the stretching of the C=O in terephthalic acid ester moiety appeared at 1724 cm^−1^. The changes in intensity observed at 1645 cm^−1^ and 1578 cm^−1^ represent the vibrations of the C=C bonds in the phenyl nucleus. The C–H bending vibrations of the CH_3_ group, symmetric and asymmetric, are observed at 1378 cm^−1^ and 1453 cm^−1^, respectively. The peak at 1274 cm^−1^ corresponds to the vibrations of the C–O bond. In the area 778–694 cm^−1^, deformation C–H vibrations outside the plane of aromatic compounds are observed.

**Figure 2:**
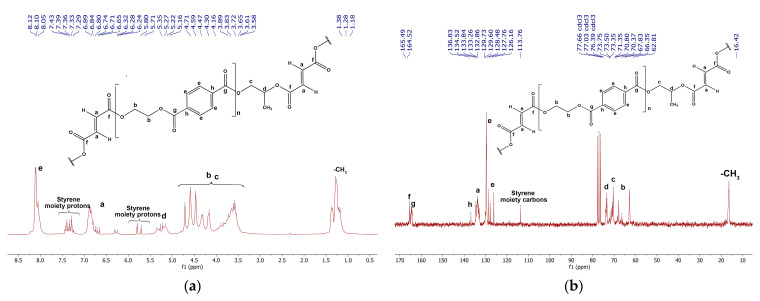
NMR spectrums: (**a**) ^1^H NMR spectrum of b-UPR resin (partially removed styrene) and (**b**) ^13^C NMR spectrum of b-UPR resin [58].

The results of ^1^H NMR (Figure 2a) and ^13^C NMR (Figure 2b) analyses indisputably confirm the success of the synthesis of b-UPR resins based on PET/PG glycolyzate. The results from a structural study of ^1^H and ^13^C NMR analyses of synthesized resin are reported below in Figure 2, respectively. The styrene from b-UPR was mostly evaporated before performing NMR experiments.

^1^H NMR (CDCl_3_, δ/ppm): 1.11–1.38 (3H, m, –CH_3_), 3.58–4.71 (14H, m, b–CH_2_ and c–CH_2_), 5.16–5.35 (1H, m, d–CH), 5.70–5.80 (2H, d, styrene moiety), 6.30–6.84 (4H, m, a–CH=CH), 7.25–7.43 (1H, m, ph-styrene moiety), 8.05–8.12 (12H, m, e–CHph). ^13^C NMR (CDCl_3_, δ/ppm): 16.42 (–CH_3_), 62.81–67.83 (b–CH_2_), 70.37–71.35 (c–CH_2_), 73.35–73.75 (d–CH), 113.76 (Cph-styrene moiety) 126.16–129.73 (e–CHph), 132.86–134.52 (a–CH=CH), 136.83 (h–Cph), 164.52 (g–C=O), and 165.49 (f–C=O).

#### 3.1.2. FTIR Analysis of Biosilica Particles and b-UPR Composites

**Figure 3:**
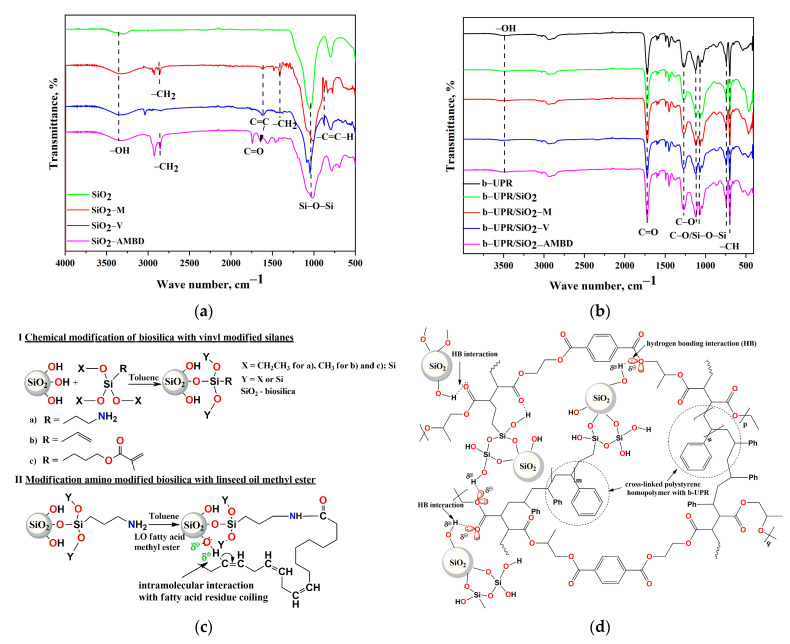
FTIR spectra of (**a**) biosilica nanoparticles and (**b**) b-UPR composites, (**c**) scheme of biosilica modification with silane and (**d**) scheme of cross-linked structure of b-UPR/SiO_2_-V composite.

In the spectra of all biosilica particles (Figure 3a), a broad band in the region from 3366 cm^−1^ to 3323 cm^−1^ comes from –OH groups stretching vibrations [59], as well as a strong peak at 1046 cm^−1^, which is assigned to Si-O-Si network vibrations, as well as siloxane stretching around 797 cm^−1^ [24,35]. The absence of organic bands in SiO_2_ proves the high purity of the biosilica obtained from the RHS. Symmetric and asymmetric vibrations of –CH_2_ groups are observed at 2930 and 2859 cm^−1^, while their bending vibrations are observed in the range from 1517 to 1370 cm^−1^ [60,61]. In the SiO_2_-AMBD spectrum, amid I vibration at 1650 cm^−1^ and N-H bending observed at 872 cm^−1^, prove successful modification with APTES [18] and subsequent modification with soybean oil fatty acid methyl ester.

Interface interactions between biosilica and b-UPR were investigated by comparison of matrix and nanocomposite spectra with 5 wt.% of nanoparticles. In all the spectra (Figure 3b), a low intensity of stretching of –OH groups are observed at ~3493 cm^−1^ [59]. Aromatic C–H stretching vibration appeared at 3027 cm^−1^, while aliphatic C–H stretching from –CH_3_ and –CH_2_ was located at 2947 and 2878 cm^−1^, respectively. The band at 1725 cm^−1^ originates from carbonyl (C=O) groups present in the terephthalic acid ester moiety [51]. Peaks at 1452 and 1371 cm^−1^ originated from CH_3_ and CH_2_ bending vibrations. Stretching vibrations of C–O appeared at 1268, 1121 and 1066 cm^−1^ [62,63]. The slight shift from 1121 to 1119 and from 1066 to 1070 cm^−1^ was observed in nanocomposite due to the overlap with Si-O-Si stretching. Aromatic –CH bending benzene rings were observed at 745 and 706 cm^−1^. The spectra of nanocomposites with lower biosilica addition showed identical bands, thus, they are not presented in this paper. The scheme of surface modification of biosilica particles with silanes is given in Figure 3c, and the scheme of cross-linked structure of the b-UPR/SiO_2_-V composite is given in Figure 3d.

#### 3.1.3. Thermogravimetric Analysis

**Figure 4:**
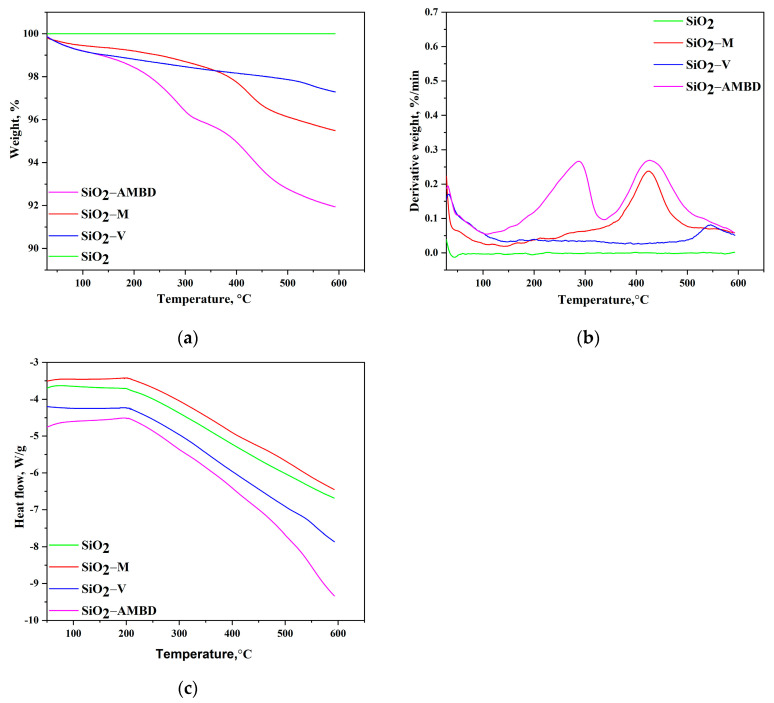
Thermogravimetric analysis of unmodified and modified biosilica particles: (**a**) TG, (**b**) dTG, and (**c**) DSC curves.

In order to investigate the difference in thermal stability between biosilica nanoparticles, TG/dTG analysis was performed (Figure 4). Unmodified silica shows stability across the entire temperature interval used. Furthermore, no surface moisture was detected [64]. SiO_2_-M shows ~1% weight loss in the temperature range between 28 and 250 °C, due to surface moisture and the evaporation of organic compounds [65]. In the second region, from 250 to 550 °C, thermal decomposition of organic modifier occurs, with the dTG peak at 424.9 °C–3.5% weight loss. The total weight loss is 4.5%. SiO_2_-V has ~1% of surface moisture lost to 100 °C. The gradual weight loss is observed until 530 °C, where a slightly sharper drop of 0.5% is observed. SiO_2_-V has approximately 2.7% weight loss. SiO_2_-AMBD has a sharp drop of 2.3% until 90 °C. In the region from 350 °C to 600 °C, there was a 3.9% loss, with a dTG peak at 427.9 °C. Total weight loss of SiO_2_-AMBD was 8.05%, which relates to the complex structure and thermal sensitivity of surface modification.

**Figure 5:**
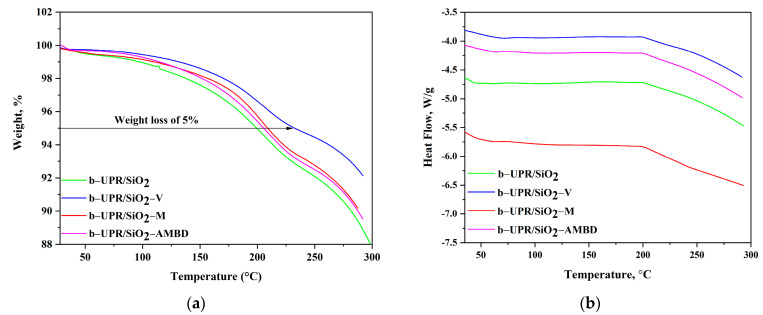
Thermogravimetric analysis of b-UPR/SiO_2_-AMBD, b-UPR/SiO_2_-M, and b-UPR/SiO_2_-V with 2.5 wt.% of particles in matrix: (**a**) TG and (**b**) DSC curves.

Thermogravimetric analysis is also used to study the influence of biosilica functionalization on the thermal properties of the b-UPR-based composites. As it is shown in Figure 5a, the weight loss that occurs at temperatures below 100 °C is due to the release of low-volatile molecules or moisture [43]. Above 100 °C, approximately 1% of weight loss is observed for b-UPR/SiO_2_ composite. Composites loaded with 2.5 wt.% of modified silica show similar thermal behavior at temperatures lower than 100 °C (Figure 5a) [43].

Thermal degradation of the b-UPR matrix above 200 °C showed ~5% weight loss. This breaking point is set to investigate the influence of biosilica functionalization on the composite’s thermal properties. The 5% weight loss is found at 199.5 °C for the b-UPR/SiO_2_ composite, while for the other composites it appeared at higher temperatures (at 232.3, 208.5, and 205 °C for b-UPR/SiO_2_-AMBD, b-UPR/SiO_2_-M and b-UPR/SiO_2_-V, respectively, see Figure 5b).

#### 3.1.4. SEM/TEM Analysis of Biosilica Fillers, b-UPR Nanocomposite and Mechanisms

**Figure 6:**
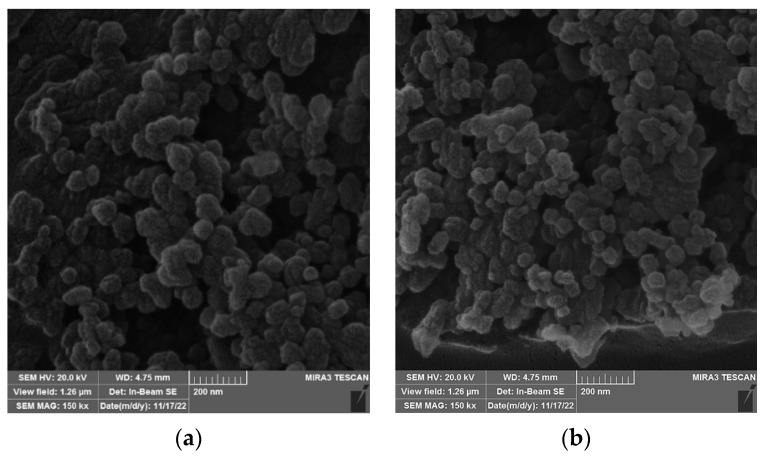
FESEM micrograph of unmodified silica particles: (**a**) unmodified, and (**b**) modified SiO_2_-V.

Field emission scanning electron microscopy (FESEM) and transmission electron microscopy (TEM) were used for studying the morphology of the unmodified biosilica (Figure 6a) and modified SiO_2_-V particles (Figure 6b). The nanostructure of silica particles after modification appears unchanged. In order to examine the dispersion, shape, and size of nanoparticles, TEM analysis was performed, which is a more suitable technique for a more detailed examination of the functionalization of nanoparticles.

The elemental analysis of the unmodified silica particles was determined using energy dispersive X-ray spectroscopy (EDX). The spectrum depicts the elemental composition of silicon dioxide particles, with silicon (31.10%) and oxygen (66.90%) dominating. This confirmed the purity of the synthesized silica nanoparticle, with a trace of elements Na, Mg, S, Cl, and Cu at 2% [42]. The diameters of the biosilica, obtained by FE-SEM micrograph analysis, ranged from 10 to 233 nm, indicating that silica particles are irregular and nanosized materials [42].

**Figure 7:**
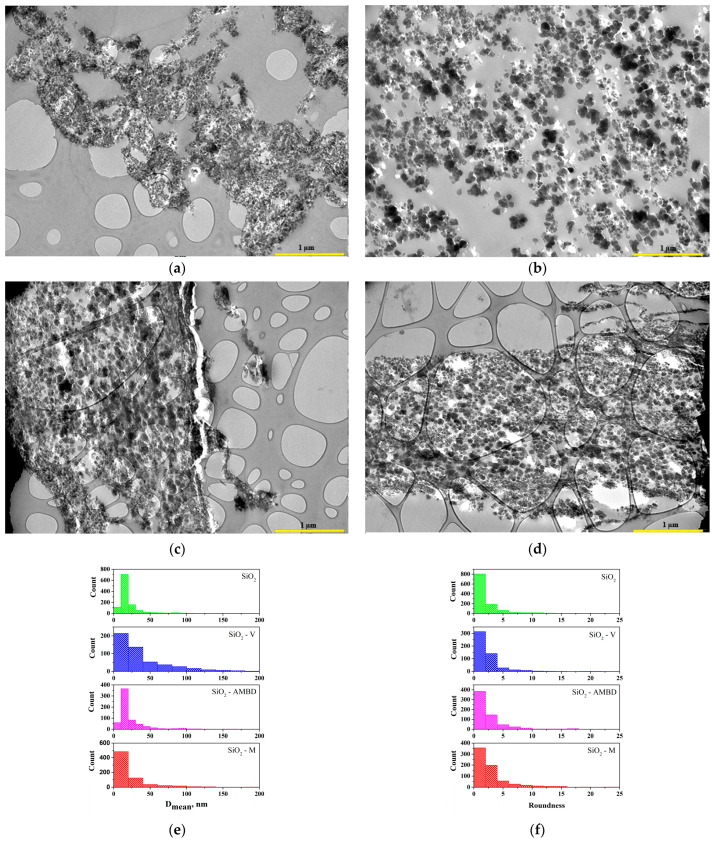
TEM micrographs of the nanocomposite with 2.5 wt.% of biosilica particles: (**a**) SiO_2_ (**b**) SiO_2_-V, (**c**) SiO_2_-AMBD and (**d**) SiO_2_-M and an image analysis of TEM aggregates present in nanocomposite with 2.5 wt.% of biosilica nanoparticles: (**e**) mean diameter, and (**f**) roundness.

Typical TEM micrographs of silica particle dispersion in polyester nanocomposite are shown in Figure 7a–d. In all samples, a chain-like structure was observed composed of partially aggregated primary spherical modified silica nanoparticles, as shown in Figure 7. In our previous work [48], an XRD analysis of silica particles was performed, and the obtained diffractogram shows a spectrum of non-crystalline solid material with no definite crystal structure, indicating that the obtained material is an amorphous fine powder. TEM analysis was used to examine the size and shape of the silica particles, as well as the uniformity and homogeneity of their distribution in the b-UPR matrix.

**Figure 8:**
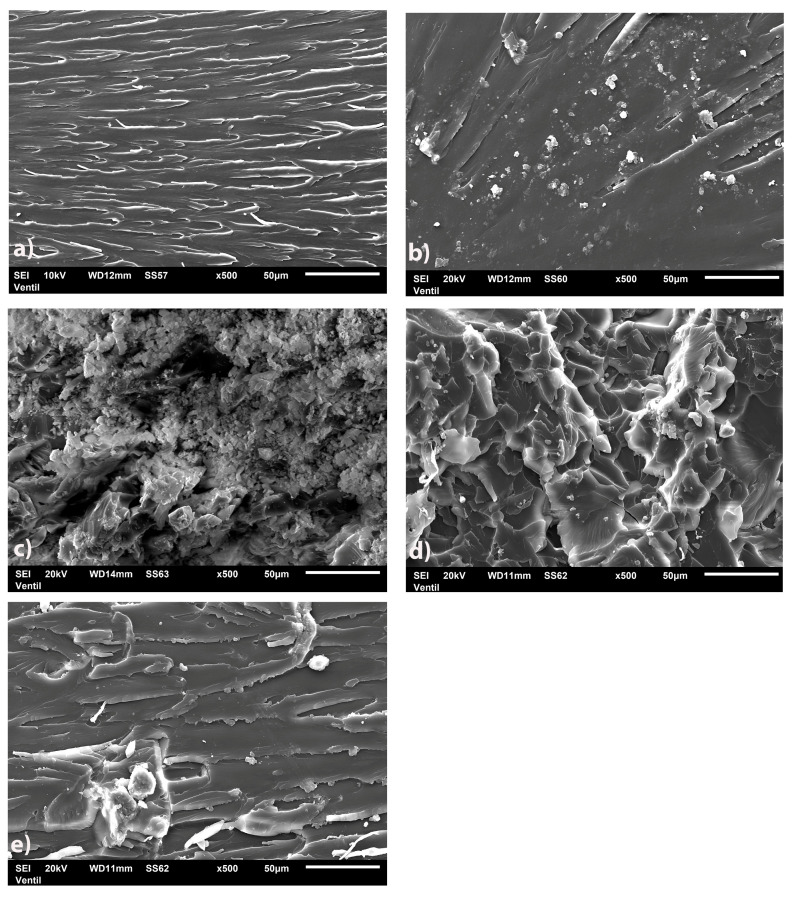
FESEM images of (**a**) polyester samples as well as nanocomposite with 2.5 wt.% of biosilica nanoparticles (**b**) SiO_2_, (**c**) SiO_2_-V, (**d**) SiO_2_-AMBD and (**e**) SiO_2_-M.

Figure 7e shows a histogram for the aggregate size distribution (*D*_mean_) of the unmodified/modified SiO_2_ nanoparticles generated by the Origin 9 software. The aggregate sphericity distribution in the polyester matrix is given in Figure 7f. Based on the histogram, the SiO_2_ nanoparticles are roughly spherical in shape, with a mean diameter ranging between 5 and 25 nm. Most aggregates in unmodified SiO_2_ and SiO_2_-AMBD are approximately 15 nm in size. The presence of agglomerates is caused by electrostatic attraction or van der Waals forces between particles [66]. Because of their small size, the obtained silica particles can be used in a variety of applications. In terms of aggregate sphericity, it can be seen that all SiO_2_ particles, both unmodified and modified, have a similar roundness. Figure 8 shows FESEM images of the fractured surface of polyester composites reinforced with 2.5 wt.% unmodified and modified biosilica.

The fractured surfaces of b-UPR and b-UPR/SiO_2_ showed similar smoothness (Figure 8a,b), while composites with modified silica particles showed higher roughness. In Figure 8c,d, it can be seen that there is undulation on the surface of the nanocomposite, which suggests that brittle fracture did not occur but that the composites have greater toughness. When the load is applied to the UPR/biosilica composites, numerous microcracks are formed, and their distribution depends on the concentration of local stress and the heterogeneity of the material. The role of biosilica particles is to reduce the stress concentration and prevent further crack propagation. Dispersion of SiO_2_-M nanoparticles was homogeneous, as can be seen in Figure 8e [48,67].

### 3.2. Mechanical Properties

The presence of vinyl reactive groups on the surface of the biosilica (Figure 3d) causes them to participate in the cross-linking reactions of curing composites based on b-UPR. The effect of several silica modifications on the mechanical properties of the generated composites, such as tensile strength, modulus of elasticity, hardness, and toughness, was investigated.

#### 3.2.1. Microhardness

**Figure 9:**
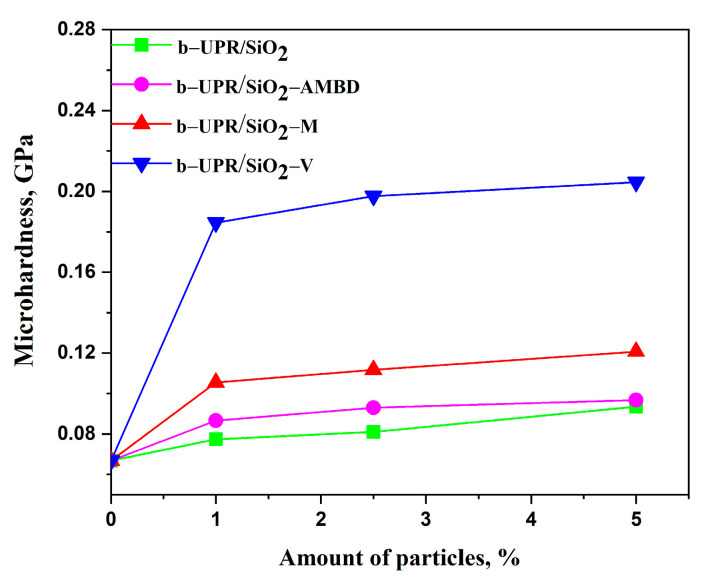
Microhardness of in b-UPR/SiO_2_ composite materials.

**Figure 10:**
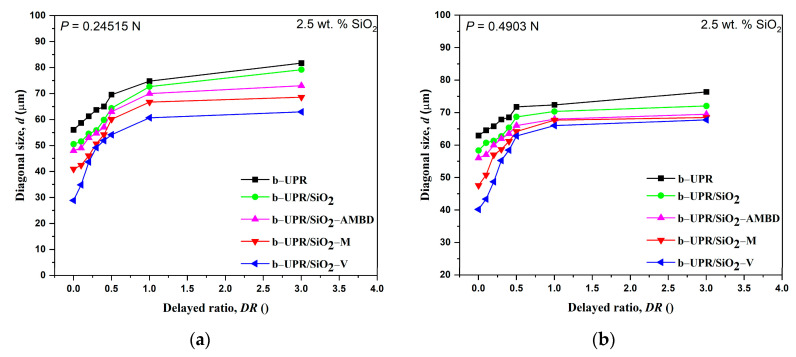

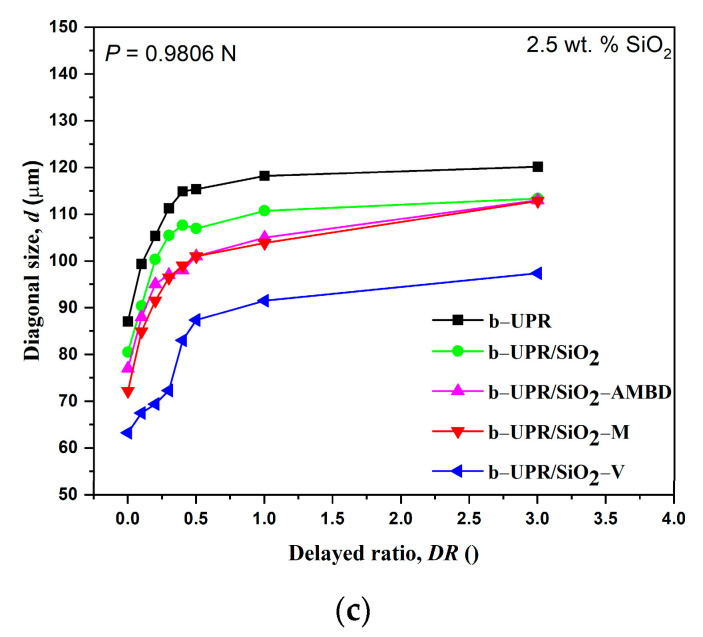
Relationship between the diagonal lengths versus delayed ratio for neat polymer matrix and composites with 2.5 wt.% of unmodified/modified silica particles. The three indentation load levels were applied: (**a**) 0.24515 N, (**b**) 0.4903 N, and (**c**) 0.9806 N.

The addition of fillers to the pure polymer matrix improves the mechanical characteristics of composite materials in general [68]. The inclusion of silica particles into the b-UPR composite boosts the hardness and strengthens the material as a result of the particles’ load-carrying capabilities. The essential difficulty in the manufacture of nanocomposite is achieving homogenous dispersion of inorganic particles in the polymer matrix to avoid aggregation and phase separation (see TEM and SEM micrographs, Figure 7 and Figure 8). Surface modification of SiO_2_ nanoparticles is performed to improve dispersion in the UPR matrix (Figure 8c–e). The determined hardness of composites depends on applied loads and the holding time of indentation, with a much greater dependency on holding time [69]. Strain and elastic recovery of the polymer material after indentation complicate the analysis. Due to the elastic recovery of the material after indentation, two separate analyses were performed. The first case represents the measurement of the microhardness of a neat matrix and composite with different types of nanosilica particles and the amount (wt.%), at a constant applied load (4.9 N) and dwell time (25 s), as shown in Figure 9. The second case of microhardness characterization of the composites is presented with the variations in delayed ratio and applied loads of constant wt.% of particles (2.5 wt.%), see Figure 10.

The comparison of microhardness values of neat matrix and composites vs. silica content (wt.%) clearly shows the extent of the silica reinforcing effect (Figure 9). The better dispersion of particles in polyester resin leads to an increase in the material’s resistance to indenter penetration, i.e., higher microhardness is a consequence. Obviously, the microhardness of nanocomposite is significantly improved with 1 wt.% of silica particles for all samples, while at higher silica addition, a low microhardness increase was observed. The optimal filler content of 2.5 wt.% was chosen due to the formation of agglomerates and micro-voids with increasing filler content. All composites have higher hardness than neat cross-linked b-UPR, except composites with 5 wt.% addition of SiO_2_-AMBD and SiO_2_ have similar values. The results prove that the silica reinforcement causes the highest hardness increase from 0.06676 GPa for the pure matrix to 0.2046 GPa for composites with 5 wt.% of SiO_2_-V addition (approximately 300% increase). The increase in microhardness values is due to the microstructural change in the composite in the course of curing and the enhanced reactivity of modified SiO_2_ particles [68]. Thus, the chemical surface modification of silica particles leads to increased intermolecular covalent bonding between the organic matrix and modified SiO_2_ particles [68].

Figure 10 shows the lengths of diagonals versus delayed ratio times for all load levels. Figure 10 shows a maximal value of strain recovery for a short dwell time (0–60 s or a delayed ratio smaller than 1). All indents were measured 100 s later, and maximal recovery was detected in the first minute after indentation (Figure 10a–c). The indents were also measured 24 h later, and there were no significant changes in diagonal size (less than 1%), which is in agreement with the reports in the literature [69,70]. The strain recovery is more pronounced at higher loads (Figure 10c) due to a larger deformation zone around the indent. A significant elastic recovery is also noticeable at a load of 0.24515 N (Figure 10a), but in that case of a small load, the change in the size of the indent can be described through the ISE effect (Indentation Size Effect) [71,72,73]. In UPR-based composites, in the presence of nanoparticles as fillers, the mechanism of viscoelastic behavior can be explained by the effect of the presence of OH groups on the SiO_2_ surface (see FTIR spectra) that act as bridges (electrostatic/dipole interactions) that inhibit the movement of the polymer chain [36]. For the other nanofillers, the main effect comes from covalent bonding in the course of UPR cross-linking, which depends on the degree of surface unsaturation as well as their availability/reactivity effectiveness. For this reason, reinforced composites have a higher resistance to elastic recovery.

**Figure 11:**
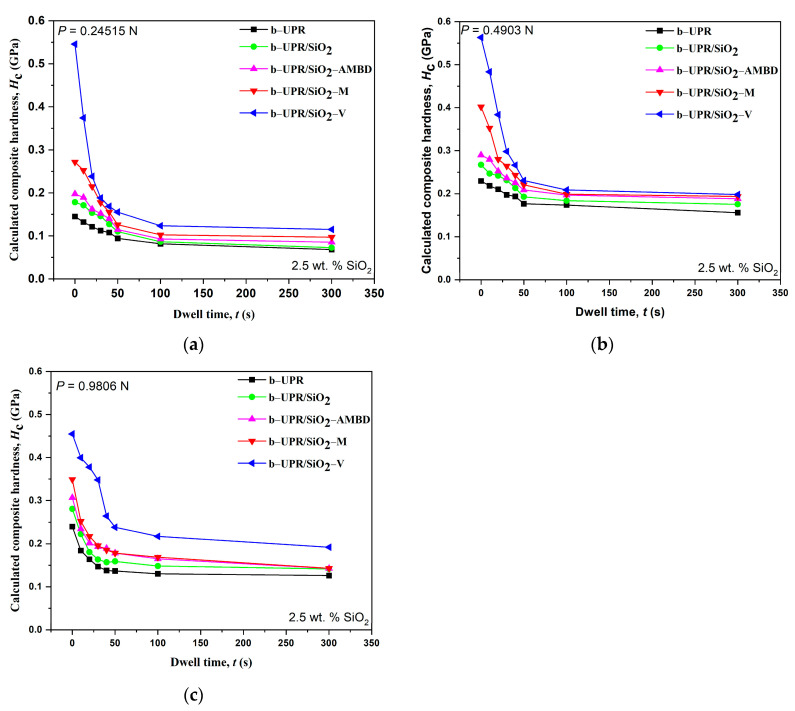
Relationship between composite hardness data and dwell time for variation samples for three load levels: (**a**) 0.24515 N, (**b**) 0.4903 N, and (**c**) 0.9806 N. The concentration of particle was 2.5 wt.%.

Figure 11 shows the calculated composite hardness *H*_c_ in [GPa] data using the experimental diagonal size measurements versus dwell time for variation modification of silica particles at constant partial concentrations (2.5 wt.%) and three load levels. It was shown that the values of composite hardness decreased as the dwell time increased. This behavior does not mean that the material is softer; it just indicates a viscoelastic response of the material affecting the hardness value [69].

As the dwell time increased to some critical value of 60 s, the hardness value decreased. By further increasing the dwell time (t > 60 s), the composite hardness does not change drastically. From the comparison of Figure 11a–c, it can be concluded that the composite hardness is less sensitive to the changes in applied loads compared to dwell time [69], and on the other hand, the composite hardness value is sensitive to the type of fillers (unmodified/modified silica particles).

Finally, the fitting parameters obtained by using the Crawford’s hardness model shown in the section characterization are displayed in Table 2. The values of parameters (*x*, *y*, and *K*) can be used to predict the hardness of omposites based on other values of applied loads and dwell times. The load is given in grams, and composite hardness is in kg/mm^2^.

Composite hardness is typically a function of many material parameters, such as yield strength, modulus of elasticity, structure, composition, type of matrix, type and concentration of filers, cross-linking type, and density. For this reason, it is difficult to generate a universal model for the prediction of hardness value as a function of applied load and dwell time. It is shown that for the same matrix of materials, the value of the hardness depends heavily on the type of filler and its concentration. For this reason, it is necessary to generate different mathematical models.

#### 3.2.2. Dynamic Mechanical Analysis

**Figure 12:**
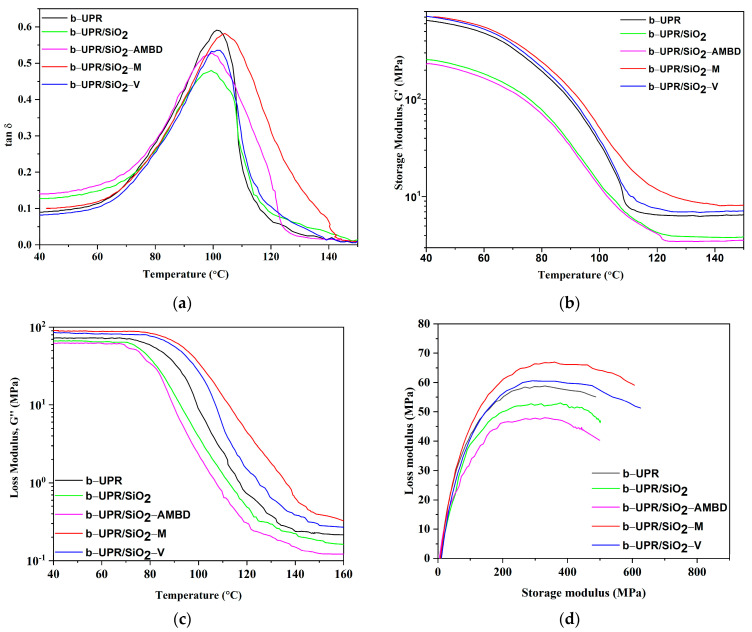
The temperature dependences of (**a**) storage modulus (*G*′), (**b**) damping factor (tan *δ*), (**c**) loss modulus (G′′), and (**d**) Cole–Cole diagram of cured b-UPR resin and biosilica-based composites.

The viscoelastic properties of cross-linked b-UPR and derived composites in the temperature range between 40 and 150 °C were investigated using DMA analysis. The temperature dependences of storage modulus (*G*′) and damping factor (tan*δ*) of cured b-UPR and biosilica-based composites versus *T* are shown in Figure 12. An increase in temperature causes a decrease in *G*′ for all samples, which is in accordance with the greater movement of the polymer segments at higher temperatures [50]. The cross-linking density (*ν*) of the cured b-UPR resin and biosilica-based composites was calculated from *G*′_RP-50°C_ according to Equation (1):

(1) $$\nu =\frac{{G^{\prime }}_{RP\text{-}50{}^{\circ}C}}{RT}$$

where *R* is the universal gas constant (8.3145 J mol^−1^ K^−1^) and *T* is *T*_g_ + 30 °C. The obtained values are shown in Table 3.

It can be noticed that composites with vinyl and MEMO silane-modified biosilica display higher values of the G′ in the whole temperature region than the neat b-UPR, and composites with unmodified and AMBD silane-modified biosilica. Those higher values of *G*′, especially on the rubbery plateau (G′RP-50 °C), indicate a higher crosslinking density (*ν*) of b-UPR/SiO_2_-M and b-UPR/SiO_2_-V composites, Table 3 [43]. Such behavior is associated with the higher strength of the interactions/chemical bonding of vinyl and methacryl reactive residues on the biosilica surface and the vinyl unsaturation present in the b-UPR polymer matrix, which further establishes the way of b-UPR chain packing and interaction (Figure 6c). The opposite is found in b-UPR/SiO_2_ and b-UPR/SiO_2_-AMBD due to the high amount of surface OH groups in unmodified SiO_2_ particles and the long fatty acid chains in BD residue which take such an orientation that disables interactions with b-UPR chains (Figure 6b). Cross-linking density significantly decreased for b-UPR/SiO_2_ and b-UPR/SiO_2_-AMBD and reached values of 40.53 and 36.85 mol·m^−3^, respectively.

The origin of the variations in the π–π stacking interaction is due to styrene and residual fragments from waste PET content, which were reflected in microhardness and G′/G′′ values. Additionally, the contribution of hydrogen bonding (Figure 3c), electrostatic attraction, and van der Waals forces are important factors that contribute to the reinforcing effect.

The tan*δ* height values are similar for neat b-UPR and composites loaded with 2.5 wt.% SiO_2_-M, while they are slightly lower for SiO_2_-V and SiO_2_-AMBD reinforced composites (Table 3). The lowest value of tanδ height is observed in the b-UPR/SiO_2_ composite (2.5 wt.% of unmodified SiO_2_). Similar values for b-UPR/SiO_2_-M and a slight decrease in tanδ height values for b-UPR/SiO_2_-V and b-UPR/SiO_2_-AMBD composites indicate that cross-linking uniformity is not diminished by introducing the reactive vinyl and methacryl surface groups [43]. The glass transition temperature (*T*_g_) is determined from the tan*δ* peak position (Figure 12b), and it reaches a value of 101 °C for neat b-UPR resin, 100 °C for both b-UPR/SiO_2_ and b-UPR/SiO_2_-AMBD, and 104 °C and 102 °C for b-UPR/SiO_2_-M and b-UPR/SiO_2_-V composites, respectively (Table 3). The increase in *T_g_* for b-UPR/SiO_2_-M and b-UPR/SiO_2_-V composites occurs due to the immobilization of the polymer macromolecular chains (restricted chain mobility), which is caused by attractive intermolecular interactions and covalent bonding between vinyl and methacryl groups on SiO_2_-V (Figure 3d) and SiO_2_-M surfaces and b-UPR chains [18]. The decrease in *T*_g_ for b-UPR/SiO_2_ and b-UPR/SiO_2_-AMBD composites occurs due to lowering the temperature of relaxation [50].

The loss modulus is a measure of the energy dissipated as heat/cycle during deformation or the material’s viscous response [74]. For composites with different biosilica modifications, the loss modulus exhibits a similar trend as the storage modulus (Figure 12a). Figure 12d shows a Cole–Cole plot of loss modulus (*G*′′) data as a function of storage modulus (*E*′) for composites containing biosilica particles. Cole–Cole plots are said to indicate polymer system homogeneity [74]. The semicircle diagram demonstrates that the polymer system is homogeneous [75]. It can be seen that different modifications of biosilica particles in the b-UPR composite change the shapes of Cole–Cole diagrams. The Cole–Cole diagram is irregular for b-UPR/SiO_2_, but semicircular for b-UPR/SiO_2_—M and b-UPR/SiO_2_—V indicating a significant dependence of the DMA properties of composites on biosilica modification.

#### 3.2.3. Tensile Strength

The homogeneous dispersion and interfacial physical/covalent interaction between biosilica particles and the polyester matrix are the keys to strengthening or toughening of the polyester matrix [31]. Furthermore, the reactive polyester matrix has good interfacial bonding with modified biosilica, mainly due to the creation of radical initiated cross-linking covalent bonds or physical entanglement (Figure 3d), resulting in the composite material being strengthened. The results of the mechanical properties of the composite, obtained according to the tensile test, are displayed in Table 4.

A comparison of modified biosilica reinforcing potential with respect to a non-modified one shows an improvement in the tensile strength of derived composites due to the increased linkage between the matrix and reinforcement material [76]. It is known that the dispersed discontinuous phase, i.e., nano-structural reinforcement, affects the physical and chemical properties of the continuous (cross-linked polyester) phase. It was also discovered that increasing the filler addition from 0 to 2.5 wt.% increases the modulus of elasticity. Tensile strength and Young’s modulus values are in accordance with TEM analysis, confirming that a higher degree of agglomeration leads to reduced mechanical performance. Additionally, a reasonable assumption about a similar trend of mechanical property change versus different concentrations of the same modified particles was confirmed [77,78]. For all of the samples, a decrease occurred with 5 wt.% of nanoparticle loads as a consequence of increased agglomeration.

Literature surveys indicate that the variation in tensile properties of UPR-based composites is significantly higher for the matrix with higher reactivity, i.e., a higher degree of unsaturation [18] or similar *σ_m_* values for composites with reactive macrofiller, itaconic acid residue-modified PET grain (PET fibers chopped to 3 mm size) used as filler [79]. In general, b-UPR/biosilica composites possess enhanced mechanical properties, in comparison to neat UPR, but they are still lower than petroleum-based ones. Tensile strength and Young’s modulus of commercial resins are in the range of 55–75 MPa, and 2 to 3 GPa, respectively. Similarly, *G*′ are lower (Table 3) than commercial (2–3 GPa), while *T_g_* is on the lower limit (commercial in the range from 100 to 150 °C). Better performances of commercial UPR arise from high crosslink density, the selection/combination of diacid, anhydride, and dialcohols used in a UPR synthesis.

In addition, the tensile test results are in accordance with the microhardness analysis, suggesting that with the presented processing method and biosilica functionalization, a sample with 2.5 wt.% SiO_2_-V shows optimal morphological and mechanical properties. The obtained results indicate the fragile properties of the studied composites at high amounts of PET-based glycolyzate. Synthesis of b-UPR, performed at a high PET:PG ratio and designed to incorporate a higher amount of waste PET, led to UPR with a structure consisting of an increased number of terephthalic acid units susceptible to effective π–π stacking interactions between the b-UPR chain and polystyrene homopolymer (obtained by the reaction of styrene as a reactive diluent in the course of curing), contributing to formation of hard segment (physical cross-linking). From one side, it could cause turbidity and the formation of precipitate, and, on the other hand, it contributes to the formation of brittle material. Thus, careful planning of stoichiometry and implementation of developed technology can be directed toward the production of materials with a minimization of negative side effects (drawbacks).

#### 3.2.4. Charpy Testing

**Figure 13:**
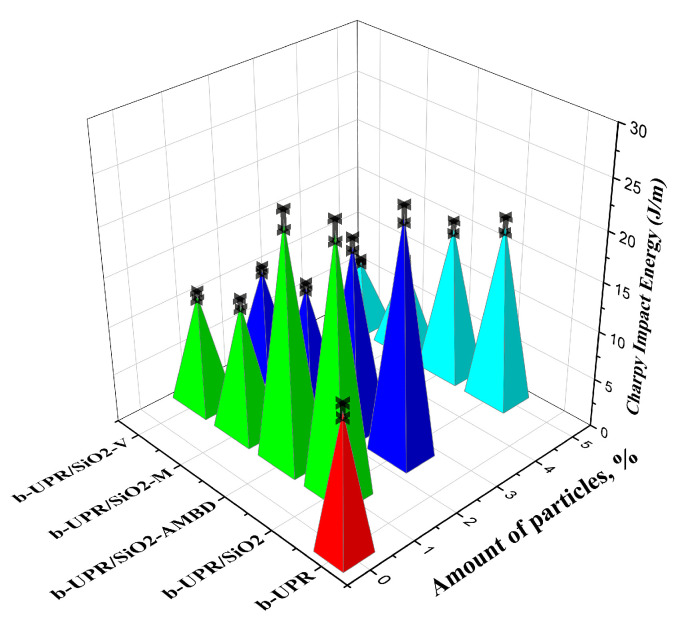
Charpy impact test of b-UPR/SiO_2_ composite materials.

Charpy’s method was used to determine the toughness of the studied nanocomposite with different amounts of silica particles and modifications, and the results are shown in Figure 13.

The maximum energy absorbed during the impact test is used to estimate the material’s toughness. When silica particles are added to the matrix, the toughness value increases, compared to the pure matrix. The addition of unmodified 1 wt.% silica particles results in the greatest increase of approximately 80.8%. Toughness begins to deteriorate after this point. This significant increase was also observed in other composite studies [80], and it can be attributed to silica cluster adsorption on polymer chains [81] as well as silica particles promoting partial stress within the matrix, which can change the direction of crack development [82]. The toughness of composite materials decreases as the filler weight percentage increases. A decrease in these values can be attributed to the formation of agglomerates, which leads to structural instability of the material, reducing the composite’s ability to absorb more energy upon impact, resulting in the formation of cracks on the surface and eventual breakage. Different surface modifications of silica particles should affect the material’s increased impact resistance [83].

### 3.3. The Extension of the Composite’s Life Cycle

After a lifespan of used b-UPR/biosilica composites, the best alternatives to extend their lifetime were found to be incorporated into a new value-added market product. The most valuable solution was found at the table top in kitchen, furniture, and floor finishing material production.

#### 3.3.1. Properties and Characterization of Table Top Based on c-UPR Resin

**Figure 14:**
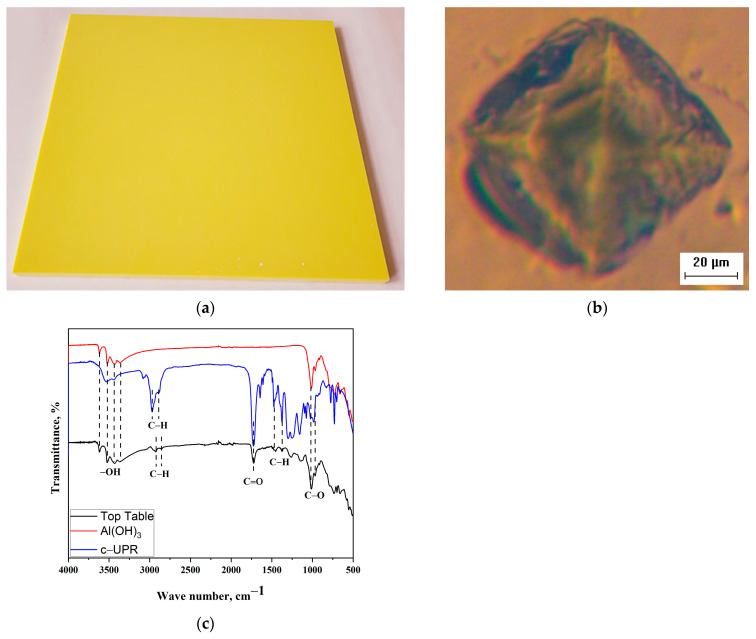
Table top material based on c-UPR (**a**), the indent obtained during the determination of the Vickers microhardness of the obtained material (**b**), and the FTIR spectra of Al(OH)_3_, c-UPR- and c-UPR-based composite (table top) with 7.9wt.% b-UPR/SiO_2_-V and 55% Al(OH)_3_ (**c**).

As described in Section 2.1.4, different amounts of composite b-UPR/SiO_2_-V-5% were incorporated in commercial table top product. The image of the upper table and floor modified composite is shown in Figure 14a; the indentation during the determination of the microhardness of the given sample is presented in Figure 14b, while FTIR spectra of the material based on c-UPR composites are shown in Figure 14c.

The hardness of the composites gradually increases as the filler content of the matrix increases. The hardening effect of the filler particles may be responsible for an increase in composite hardness [84]. The microhardness of table top and floor material based on c-UPR composites is 0.361 GPa (19.9 wt.% b-UPR/SiO_2_-V and 42 wt.% Al(OH)_3_) and 0.373 GPa (7.9 wt.% b-UPR/SiO_2_-V and 55 wt.% Al(OH)_3_). The resulting microhardness value is almost double the value obtained from bio-renewable sources.

The band in the region 3614–3355 cm^−1^ corresponds to the stretching vibration of residual –OH groups. The bands around 2921 cm^−1^ and 2853 cm^−1^ arise from aliphatic C–H stretching vibration. The C=O stretching vibration of the carbonyl group appeared at 1726 cm^−1^. Asymmetric and symmetric bending vibrations of the methyl C–H in-plane were observed at 1449 cm^−1^ and 1373 cm^−1^, respectively. C–O asymmetric stretching appeared at 1150 cm^−1^ and 1014 cm^−1^, respectively. Out-of-plane aromatic C–H vibrations were observed in the region 730–662 cm^−1^ (see Figure 14c).

**Figure 15:**
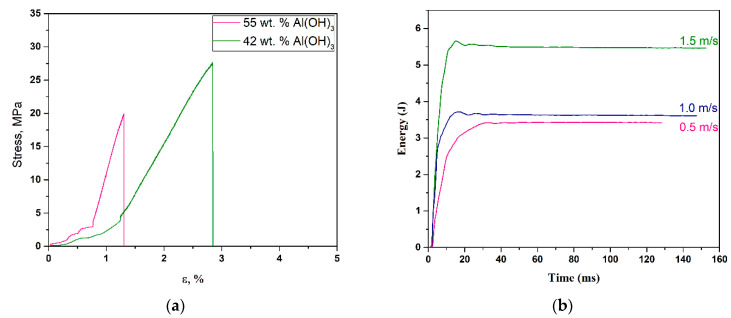
Stress vs. strain curves for c-UPR-based composite with 42 wt.% and 55 wt.% Al(OH)_3_ (**a**) and the energy–time curves obtained from impact test for table top based on c-UPR composites with 55 wt.% Al(OH)_3_ (**b**).

Figure 15a shows tensile strength results for c-UPR/Al(OH)_3_ composites. The time-dependent impact properties of the composites filled with 55 wt.% Al(OH)_3_ are shown in Figure 15b. According to Wu et al. [85], the tensile strength of the UPR/Al(OH)_3_ composite increased with increasing Al(OH)_3_ content, reaching a maximum value of 20 wt.% Al(OH)_3_ content. The tensile strength and elongation at the break of the c-UPR/Al(OH)_3_ composite then decreased as the particle proportion increased. The increase in absorbed energy can be associated with the difference in mechanisms of energy absorption depending on the impact speed [86].

#### 3.3.2. The Results of the Flammability Test

Because of its low cost and effectiveness, (Al(OH)_3_) is the most commonly used fire retardant. When heated to approximately 220 °C, Al(OH)_3_ decomposes into aluminum oxide (Al_2_O_3_) and water. As a result, Al(OH_)3_ acts as a heat sink by releasing water vapor in an endothermic process, which accounts for approximately 35% of its weight [87].

Three times-repeated tests were conducted in accordance with the guidelines outlined in the UL-94 standard [53] vertical test to evaluate the thermal stability of the table top based on c-UPR/Al(OH)_3_ composites. Following ignition of the composite, the test measures the material’s propensity to either extinguish or spread the flame. Four categories—NC, V-2, V-1, and V-0—can be applied to evaluate the fireproofing properties of the samples. Due to the fast extinguishment of the flame and low material charring, the final image in the series demonstrates the sample’s resistance to flame. The best rating, V-0, has been assigned to the c-UPR/Al(OH)_3_-based composite. When the burner was taken away, the sample immediately went out, and 5 s were recorded after the second exposure to the flame. Additionally, according to Seraji et al. [88], the sample of UPR incorporated with 40 wt.% of Al(OH)_3_ has reported a V-0 rating in the UL-94 fire test.

## 4. Conclusions

In conclusion, the most valuable achievements of this work are given, as follows:

- A method for producing b-UPR from waste PET glycolyzate and biobased PG and MAnh was presented.
- Rice husk (RH) silica particles were nanostructured and then modified with various vinyl silanes before being employed as reinforcement in b-UPR-based composites.
- The structural, morphological and mechanical properties of b-UPR and the obtained composites were investigated.
- Silica, incorporated into b-UPR resin, formed chain-like aggregates and covalent bonds in the course of cross-linking with the b-UPR matrix.
- The highest *σ*_m_ of 88% was obtained for 2.5 wt.% of SiO_2_-V addition in b-UPR/SiO_2_-V. Furthermore, microhardness increased by more than 200%, from 0.0668 GPa, found for UPR, to 0.205 GPa for UPR/SiO_2_-V (5 wt.%)
- Different modifications of SiO_2_ nanoparticles showed a certain influence on the dynamic-mechanical properties of the obtained nanocomposite. *T_g_* was not significantly affected by the presence of SiO_2_, showing a neglectable increase for b-UPR/SiO_2_-M and b-UPR/SiO_2_-V composites, 104 °C vs. 102 °C, compared to 101 °C obtained for neat b-UPR.
- Grounded b-UPR/SiO_2_-V composite was used as filler for the production of c-UPR-based table top and floor materials.
- The test results of the table top material showed outstanding mechanical properties: microhardness of 0.361 GPa (42 wt.% Al(OH)_3_) and 0.373 GPa (55 wt.% Al(OH)_3_), as well as fire-resistant properties rated in category V- 0 according to the UL-94 method.
- The principles of green chemistry are implemented through minimization of waste generation, use of renewable raw materials, greener product design and minimization of solvent use.
- The continuation of the research will be devoted to the synthesis of fully biodegradable UPR, using vinyl reactive lignin and tannic acid, in order to improve fireproofing properties without addition of commercial flame-retardant additives.

## Figures and Tables

**Table 1:** Physicochemical properties of b-UPR resin.

Parameter	Value	Unit	Method
Appearance	Transparent yellow	-	-
Non-volatile material content	60 ± 2	wt.%	ISO 3251
Acid/Hydroxyl value (AV/HV)	12/25	mg KOH/g	ISO 2114
Gel time, 20 °C: 2 wt.% MEKP-50	25–30	Min	ISO 2535
Temperature of exotherm peak	110–120	°C	ISO 2535
Density	1.37 ± 0.09	kg/m^3^	ISO 2811
Iodine value	51	-	Wijs

**Table 2:** The optimal fitting parameters for prediction b-UPR composite (with 2.5. wt.% of silica particles) hardness; *n*_av_—an average slope, *A* and *B* are fitting constants for calculation *K*, *x*, and *y* Craford’s parameters.

Sample	*n*_av_	*A*	*B*	*x*	*y*	*K*
b-UPR	2.2499	0.0060	−0.1122	0.1111	−0.0997	19.6738
b-UPR/SiO_2_	2.2367	0.0121	−0.2544	0.1406	−0.2275	35.7390
b-UPR/SiO_2_-AMBD	2.3178	0.0078	−0.2231	0.1371	−0.1952	28.1794
b-UPR/SiO_2_-M	2.1038	0.1024	−0.6910	0.0493	−0.6569	21.2490
b-UPR/SiO_2_-V	2.6520	0.0214	−0.9776	0.2458	−0.7373	48.0749

**Table 3:** The values of the *G*′_RP-50°C_, *G*′_GS-130°C_, *T_g_*, tan *δ*_height_ and *ν* for cured b-UPR resin and biosilica-based composites.

Sample	*G*′_RP-50°C, MPa_	*G*′_GS-130°C, MPa_	*T*_g_, °C	tan *δ*_height_	*ν ×* 10^−3^, mol m^−3^
b-UPR	581.86	6.35	101	0.59	103.37
b-UPR/SiO_2_	227.83	3.91	100	0.48	40.53
b-UPR/SiO_2_-AMBD	207.12	3.47	100	0.53	36.85
b-UPR/SiO_2_-M	658.36	9.28	104	0.58	116.44
b-UPR/SiO_2_-V	640.05	6.98	102	0.53	113.53

**Table 4:** Mechanical properties of b-UPR composites.

Sample	SiO_2_ Amount (wt.%)	*σ*_m_ (MPa)	*E* (GPa)	ε (%)
b-UPR	-	25.5 ± 1.11	0.276 ±0.013	5.90 ± 0.25
b-UPR/SiO_2_	1	28.7 ± 1.33	0.316 ± 0.015	5.58 ± 0.18
b-UPR/SiO_2_-AMBD	1	34.3 ± 1.54	0.345 ± 0.016	5.35 ± 0.24
b-UPR/SiO_2_-M	1	38.4 ± 1.77	0.351 ± 0.018	5.22 ± 0.21
b-UPR/SiO_2_-V	1	43.6 ± 1.97	0.412 ± 0.019	4.57 ± 0.16
b-UPR/SiO_2_	2.5	32.1 ± 1.41	0.412 ± 0.020	5.41 ± 0.18
b-UPR/SiO_2_-AMBD	2.5	39.8 ± 1.69	0.490 ± 0.024	4.97 ± 0.26
b-UPR/SiO_2_-M	2.5	40.1 ±1.82	0.558 ± 0.027	4.81 ± 0.23
b-UPR/SiO_2_-V	2.5	47.9 ±2.18	0.607 ± 0.029	4.02 ± 0.21
b-UPR/SiO_2_	5	28.2 ±1.35	0.285 ± 0.014	5.56 ± 0.12
b-UPR/SiO_2_-AMBD	5	27.0 ± 1.31	0.316 ± 0.015	5.78 ± 0.22
b-UPR/SiO_2_-M	5	38.9 ± 1.82	0.394 ± 0.019	5.06 ± 0.19
b-UPR/SiO_2_-V	5	41.7 ± 1.91	0.378 ± 0.018	4.73 ± 0.23

*σ*_m_—tensile strength, *E*—Young’s modulus, and *ε*—deformation.

## Data Availability

The data presented in this study are available on request from the corresponding author or co-authors. The data are not publicly available.
